# Prevalence and factors associated with suicidal ideation, cannabis, and alcohol use during the COVID-19 pandemic in Saskatchewan: findings from a joint-effect modeling

**DOI:** 10.1186/s12888-023-05051-w

**Published:** 2023-08-08

**Authors:** Daniel A. Adeyinka, Nuelle Novik, Gabriela Novotna, Mary Bartram, Robert Gabrys, Nazeem Muhajarine

**Affiliations:** 1 Saskatchewan Population Health and Evaluation Research Unit (SPHERU), University of Saskatchewan, 104 Clinic Place, Saskatoon, SK S7N 2Z4 Canada; 2 Department of Community Health and Epidemiology, College of Medicine, University of Saskatchewan, 107 Wiggins Rd, Saskatoon, SK S7N 5E5 Canada; 3 Faculty of Social Work, University of Regina, 3737 Wascana Parkway, Regina, SK S4S 0A2 Canada; 4 School of Public Health and Administration, Carleton University, Ottawa, ON K1S 5B6 Canada; 5 Mental Health Commission of Canada, Ottawa, ON K1R 1A4 Canada; 6 Canadian Centre On Substance Use and Addiction, Ottawa, ON K1P 5E7 Canada

**Keywords:** Suicidal ideation, Cannabis use, Alcohol use, COVID-19, Pandemic, Saskatchewan

## Abstract

**Background:**

Generally, pandemics such as COVID-19 take an enormous toll on people’s lives. As the pandemic now turns to an endemic state, growing attention has been paid to the multiple adverse mental health and behavioral issues, such as suicidal ideation and substance use. However, the interplay of suicidality and substance misuse during the pandemic has been limited. We aimed to investigate the prevalence of co-occurrence of suicide ideation, alcohol and cannabis misuse, and the factors that are associated with these co-occurrences in the province of Saskatchewan during the COVID-19 pandemic.

**Methods:**

We performed a multivariable trivariate probit regression on a sample of 666 Saskatchewan adolescents and adults (16 years or older), drawn from the cycle 10 data collection (March 2022) of the Mental Health Commission of Canada, and Canadian Centre on Substance Use and Addiction (MHCC-CCSA) dataset.

**Results:**

The prevalence of suicidal ideation was higher among respondents who reported both problematic cannabis and alcohol use (25.8%) than single users of alcohol (23.2%) and cannabis (18.7%). Younger respondents (16–34 years) and those who reported recent changes in other substance use were independent factors that were associated with the common experience of suicide ideation, problematic cannabis, and alcohol use. Having a diagnosis of mental health disorders either before or during the pandemic, and the perceived inability to bounce back after the pandemic (low resilience) are strong correlates of suicidal ideation. Those who lived alone, between 35 and 55 years of age were more likely to report problematic alcohol use. Those who reported changes in alternative activities, who reported pandemic stress, and declared a LGBTQIA2S + identity had higher probability of problematic cannabis use.

**Conclusions:**

As the pandemic persists, improving access to suicide and substance use interventions for the vulnerable groups identified in this study may be impactful.

**Supplementary Information:**

The online version contains supplementary material available at 10.1186/s12888-023-05051-w.

## Introduction

Globally, suicide and substance use are prevalent public health issues. Many have speculated that these issues have been exacerbated due to the impact of COVID-19 pandemic [1]. Some people, though, have opined that suicide rates tend to decline during crises and natural disasters previously, a “pulling together phenomenon” wherein people tend to care more for each other during emergency situations [2, 3]. This phenomenon is complemented by the interpersonal theory of suicide [4, 5], which posits that suicidal desire is a result of two interpersonal forces at work simultaneously—thwarted belongingness and perceived burdensomeness [6].

Within Canada, suicide is the ninth leading cause of death in the general population, and the second leading cause of death among people between 15 and 34 years of age [7]. The prevalence of suicide and substance use vary considerably across the provinces and territories. In 2021, Saskatchewan had 13.8 deaths by suicide per 100,000 population; one of the highest among the provinces and territories in Canada [8]. Substance use independently increases the risk of suicide [9]. Furthermore, people who experience problematic substance use are more vulnerable to severe medical complications such as liver cirrhosis, vehicle accidents, pneumonia, HIV, homicide, cardiovascular disorders, especially coronary heart diseases, and malignancies [10, 11].

Alcohol use is the most common addiction in Canada, followed by cannabis use [12]. When consumed excessively, both substances can have health and social consequences. According to the 2020 report by the Canadian Centre on Substance Use and Addiction (CCSA), premature deaths and disabilities from substance use resulted in loss of productivity and had major impacts on the Canadian economy [13]. Psychoactive substances are often consumed for pleasure or to relieve stress [14]. In times of crisis, like the COVID-19 pandemic, increasing reliance on alcohol and cannabis has been partly attributed to concerns about being sick, caring for sick family members, loss of job, economic downturn, bereavement, boredom, and stress due to the pandemic. According to the 2022 MHCC/CCSA report [7], one in four deaths by suicide was recorded among those who over-used alcohol. Problematic alcohol use has been ranked as the second most common mental health problem in people who died by suicide, after depression [7]. Uncontrolled alcohol use can increase suicide risk through different mechanisms, which include dampening of one’s fear, disinhibition, impairment of judgement, impulsivity, constriction of cognition, and increased psychological distress and aggressiveness, especially when one is intoxicated. Although the impact of other substances (such as cannabis) on suicidality remains largely unknown because of the conflicting evidence [15, 16], literature suggests that the number of substances used is more important in predicting suicidality than the type of substances used [17]. It is generally known that simultaneous use of both cannabis and alcohol has additive harmful effects than using either substance on neuropsychological functioning [18, 19].

Suicide is a complex phenomenon, which is not fully understood [20, 21]. According to the Mental Health Commission of Canada (MHCC), suicide is a sequel of a gradual build-up of overwhelming emotional and physical discomforts in the face of low resilience (state of hopelessness), which is often associated with factors such as family history of suicide, sociocultural factors, stressful events, and neurobiological factors [21].

Early evidence from studies conducted in Canada during the pandemic highlighted that some public health countermeasures implemented to curb community transmission of COVID-19 such as social distancing and closure of economic activities could have predisposed people to suicidal ideation and substance use [21]. During the first year of the pandemic, Canadians who reported experiencing suicidal thoughts/feelings due to the pandemic ranged from 4.2% to 8.3%, which was significantly higher than the 2.7% in 2019 (pre-pandemic) [22–24]. It was noted that social distancing and limited social interactions also aggravated pre-existing mental health conditions in part due to the disruption in healthcare system [21]. Evidence suggests that the pandemic has been accompanied by an increase in substance use [25], especially among people with past and current mental health issues [23, 26–28]. Within Canada, there were variations in how the provinces and territories responded to COVID-19, which have short- and long-term consequences. As part of the Saskatchewan’s health response, non-emergency services such as elective surgeries, diagnostic procedures, home care, rehabilitation, diabetes counselling and outpatient appointments were deprioritized to free up resources for COVID-19 patients and reduce transmission risks. The deprioritization led to a backlog of patients and increased wait times for non-COVID-19 healthcare, hence creating additional challenges to accessing essential care [29–31]. A recent repeated cross-sectional study in Saskatchewan found a stable trend, but somewhat elevated prevalence of depression and anxiety since onset of the pandemic [32]. Most strikingly, mental health supports were inaccessible to most Saskatchewan adults who needed them [32]. Much remains unknown about the impact of the pandemic on mental health issues such as suicidal ideation and substance use in Saskatchewan.

Studies have indicated that suicidal risk is not evenly distributed across the population, due to structural and social factors [23, 27, 28, 33]. Of particular interests are the equity-deserving populations (e.g., Indigenous people, visible minorities, those who declare LGBTQIA2S + , and people living with disabilities), who were two to four times more likely to have had suicidal thoughts since the outbreak of COVID-19 [21, 23, 28]. Overall, men are more likely to report suicidal ideation [7] or substance use [34], although there have been recent reports of increasing prevalence of substance use in both men and women. Also, people who experience problematic cannabis and alcohol use are particularly vulnerable to suicidal ideation, and ultimately suicide, when faced with socioeconomic, and health challenges [15, 35, 36]. Elevated alcohol and cannabis use, in the presence of suicidal thoughts, are harmful coping behaviors which could lead to death if intervention is not promptly implemented.

The determinants of suicidal ideation, alcohol, and cannabis use have been investigated individually [37–39], but little is known about the inter-relationship between these three health conditions especially during the pandemic. In this paper, we aimed to investigate the prevalence of co-occurrence of suicide ideation, alcohol and cannabis use, and the factors that are associated with these co-occurrences during the COVID-19 pandemic (in March 2022). We hypothesized that some factors will be associated uniquely with different combinations of the three outcomes, while also expecting some other factors to be associated commonly with these three outcomes.

## Methods

### Study setting

Saskatchewan is a landlocked province in western Canada that is bordered on the south by the United States, west by Alberta, north by Northwest Territories, east by Manitoba, and northeast by Nunavut. According to the Statistics Canada, Saskatchewan’s population for 2021 was 1.13 million, translating to 3.1% of Canadian population [40]. About 75% of the Saskatchewan residents live in the cities and towns, clustered in the southern prairie. The northern part of the province is sparsely populated. Women accounted for 51% of the total population. Saskatchewan had 41% of its population aged 55 years and over.

### Study sample and design

This is a cross-sectional survey, conducted by Leger research firm on behalf of the Canadian Centre on Substance Use and Addiction (CCSA) and the Mental Health Commission of Canada (MHCC), to progressively monitor the impact of the COVID-19 pandemic on the mental health and substance use in Canada since October 2020. The MHCC-CCSA survey consists of a series of bimonthly population-based study that is administered to Canadian residents who are 16 years and older via self-administered, and autonomous web-based panel survey (i.e., Computer Aided Web Interviewing (CAWI) technology). The internet panel comprises of 400,000 Canadians across the provinces and territories who were primarily selected through a hybrid recruitment approach—random selection and recruitment from a self-selected sample [41, 42]. Most Leger’s panel members (61%) have been recruited randomly over the phone over the past decade and profiled on different sociodemographic variables (geographical region, age, gender, immigration status and ethnicity).

Respondents for the surveys were selected from among those who have volunteered and registered to participate in online surveys. To ensure robustness of the samples that approximate the true population parameters, quotas were implemented and the amount of sample to be drawn was adjusted based on the historical response rate of each demographic sub-group, with a sample size equivalent to a probability (random) sampling of ± 2.5% margin of error [43].

For this study, the French and English survey instruments were pre-tested with a small sample of respondents to ensure the survey material was easily understood by respondents, and that the resultant data were being captured properly. We analysed participants’ responses from the cycle 10 data collection (March 2022), focusing on Saskatchewan samples. We partnered with MHCC-CCSA in cycle 10 to draw an over-sample of Saskatchewan respondents. The cycle 10 data collection phase coincided with a downward trend in COVID-19 incidence, hospitalizations, and deaths, and easing of public health measures across the provinces and territories of Canada. During March 2022, there was a slow but steady growth in the BA.2 sub-lineage of the Omicron variant in Canada [44].

The study sample consisted of 666 Saskatchewan adolescents and adults (aged 16 years and older) who responded to the questions on suicidal ideation, alcohol and cannabis use during the late stage of the second year of the pandemic.

### Measures

#### Dependent variables

The dependent variables were: (1) suicidal ideation (2) intensity of problematic cannabis use, and (3) intensity of problematic alcohol use. Suicidal ideation was assessed by directly asking the respondents if they had seriously contemplated suicide since the COVID-19 pandemic began in March 2020. The responses were dichotomised into no (reference category) and yes.

The Cannabis Use Disorder Identification Test-Revised (CUDIT-R), an 8-item standardized tool was used to measure the intensity of problematic cannabis use by screening participants for symptoms of cannabis dependence over the past 6 months. The CUDIT-R tool covers 4 domains of cannabis consumption, experience of cannabis-related harms, potential dependence on cannabis, and psychological aspects [45]. A Cronbach’s alpha of 0.83 was obtained for this sample, implying a good scale reliability. As pre-defined in the literature [45], we constructed a dichotomous variable that defines a score of ≥ 8 as “problematic or hazardous” cannabis use; otherwise as “abstainers or low risk consumers” (reference category).

The third outcome, intensity of alcohol use was assessed using a 10-item form of the Alcohol Use Disorders Identification Test (AUDIT). This self-screening questionnaire was designed by the World Health Organization to measure alcohol consumption, drinking behaviors, and alcohol-related problems in the past year [46]. The screening tool was modified to capture the drinking patterns over the past 6 months to minimize recall bias. For this study, the Cronbach’s alpha was 0.89, indicating a good scale reliability. Each response has a score ranging from 0 to 4. All the response scores were summed for a total score. For AUDIT, scores could range from 0 to 40. As predefined in the literature [46], the variable on alcohol use was dichotomized, a score of 8 or more indicating “problematic or hazardous” alcohol use, and less than 8 indicating “low risk or abstainers” (reference category).

#### Independent variables

The independent variables are described in Table 1. They are divided into 4 broad categories, namely: sociodemographic characteristics, mental health status, adaptability, and accessibility to formal treatment services. The independent variables were selected a priori based on previous literature and data availability.

**Table 1:** Operational definition of independent variables

Categories	Independent variables
Sociodemographic characteristics	Age (16–34, 35–54, ≥ 55 years old)
	Gender (Woman, man)
	Highest education (High school or less, college, university)
	Household income (≤ $20 k, $21 k to $50 k, $51 k to $100 k, > $100 k)
	Ethnicity (White, BIPOC)
	Gender and sexual identity: self-declaration of LGBTQIA2S + identity (No, yes)
	Household composition (Living alone, Living with others)
	Migration status (Canadian-born, immigrant)
	Place of residence in Saskatchewan (Regina, Saskatoon, north, central, south)
Mental health status	Diagnosis of mental health disorder? (No, yes-before pandemic, yes-during pandemic)
	COVID-19 related or pandemic stress (No, yes)
Adaptability	Resilience^a^: ability to bounce back after pandemic is over (grouped into low (< 3) or normal/high (≥ 3))
	Recent changes in coping behaviors (2 subscales generated from 11-items using factor analysis, see appendix A) i. Changes in other substance use (median classification: low, high) ii. Changes in alternative activities (median classification: low, high)
Accessibility to formal treatment services	Mental health services (not needed and not accessed, needed but not accessed, needed and accessed)
	Cannabis addiction services (Not applicable, no, yes)
	Alcohol addiction services (Not applicable, no, yes)

^a^Brief Resilience Scale [47]

Participants’ resilience (i.e., perceived ability to bounce back after pandemic) was assessed using the Brief Resilience Scale (BRS) [47]. The details of the items in the scale could be seen elsewhere [47]. It is a 6-item scale which consists of questions relating to perceived ability to recover from stress. Responses were provided using 5-point Likert scale, ranging from 1 (strongly disagree) to 5 (strongly agree) for the positively worded questions (items 1, 3 and 5) but reverse coded for the negatively worded questions (items 2, 4 and 6). The Cronbach’s alpha is 0.89, indicating an acceptable level of internal consistency. A prorated score was derived by summing the responses from the items and dividing the total scores by the number of questions answered. As pre-defined in literature [47], the level of resilience was classified as low if the BRS score is between 1 and 2.99 and normal to high resilience if the score is between 3 and 5.

To determine how people are adjusting to the pandemic, we assessed the recent changes in coping/self care behaviors adopted by the respondents. The respondents completed a 19-item instrument, designed by the survey team to assess the changes in lifestyle behaviors with a 4-point Likert scale ranging from 1 (not applicable) to 4 (far more). The instrument has an acceptable internal consistency with a Cronbach’s alpha of 0.74. The instrument is fully described in the Appendix A. The two items on alcohol (item 14) and cannabis consumption (item 15) were dropped because their underlying constructs were empirically measured by the outcome variables of interest. With principal factor analysis, 6 redundant items which weakly loaded on the factors were eliminated. In the re-specified model, the dimensionality of the remaining 11 items were further reduced to 2 subscales (i.e., the first 2 factors which accounted for the total variance). The extraction was based on Kaiser criteria–Eigenvalue ≥ 1, Kaiser–Meyer–Olkin measure of sampling adequacy ≥ 0.5, Bartlett’s test of sphericity < 0.001. Following the guidelines for items retention based on a sample size of more than 350 respondents [48], we ensured discriminant and convergent validity by selecting items that substantially loaded on a factor if the absolute loading score is ≥ 0.3 on an orthogonal varimax rotation with Kaiser normalization. The first factor, labelled as changes in other substance use, loaded substantially on other forms of substance use such as cigarette smoking, vaping, prescribed psychoactive drugs (e.g., Ativan, Xanax, Dilaudid, etc.– excluding cannabis), and other illegal psychoactive drugs (e.g., cocaine, opioids, methamphetamine, etc.). The second factor, identified as alternative activities, was typified by physical activities, relaxation activities (e.g., mindfulness meditation, hot bath), connection with friends and family, work engagement and sexual intimacy). The scores were further recoded into two categories based on the median values: low and high levels. The scores were dichotomized to ease interpretation of results, allowing for comparison in the relative effects between the low and high categories. Also, the changes in coping behavior during the pandemic were not homogenous. Some studies have emphasized the need to dichotomize continuous variables in certain circumstances such as behavioral and mental health studies [49–51]. The other covariates considered and how they were coded are provided in Table 1.

### Statistical analysis

Descriptive analyses were performed and expressed as frequencies and percentages. In addition, a Venn diagram of the outcome variables was generated to determine the degree of intersectionality between suicidal ideation, and problematic cannabis and alcohol use. The bivariable relationships between each outcome variable and explanatory variables were tested with Chi-square test or Fisher's exact test, as appropriate. The pairwise associations among the outcome variables were assessed using tetrachoric correlation with Bonferroni-adjusted significance levels. Based on the observed significant associations from the tetrachoric correlation, modeling with a trivariate probit regression was justified as appropriate for accounting for the common unobservable factors than fitting separate models for the outcome variables [52]. On this note, we implemented a multivariable trivariate probit regression to concurrently fit the relationship between three binary outcomes–suicidal ideation, alcohol and cannabis use while looking for factors affecting the three conditions. Additionally, this approach allowed for each dependent variable to serve as covariate. The marginal effect is the predicted change in probability of the dependent variable for a unit change in the explanatory variable. The regression coefficients of the independent variables were interpreted as the changes in probability of the dependent variables when the values of marginal effects were equivalent to the coefficients [53, 54]. In this regard, we interpreted the coefficients as marginal effects (i.e., probabilities) because both estimates were the same. To prevent over-fitting, the independent variables were selected into the multivariable model based on a *p*-value ≤ 0.2, however, the variables on the access to treatment were dropped because of multicollinearity issues. Using a stepwise elimination approach, the variables with the highest *p*-values were dropped until a parsimonious model with the lowest Akaike information criterion (AIC) and Bayesian information criterion (BIC) was achieved. We ensured model stability by setting the number of random draws at 26, which is the square root of the sample size [52]. The level of statistical significance was set at *p*-value < 0.05. Data analyses were implemented in Stata version 17.0 [55].

## Results

### Characteristics of respondents

The sample characteristics of the respondents are presented in Table S1. Most of the respondents were 55 years or older (58.7%), women (57%), living with other people (73.8%), non-immigrant (89.6%), and had experienced COVID-19 related stress (83.2%).

#### Prevalence of suicidal ideation, problematic cannabis, problematic alcohol use and co-occurrences

**Fig. 1:**
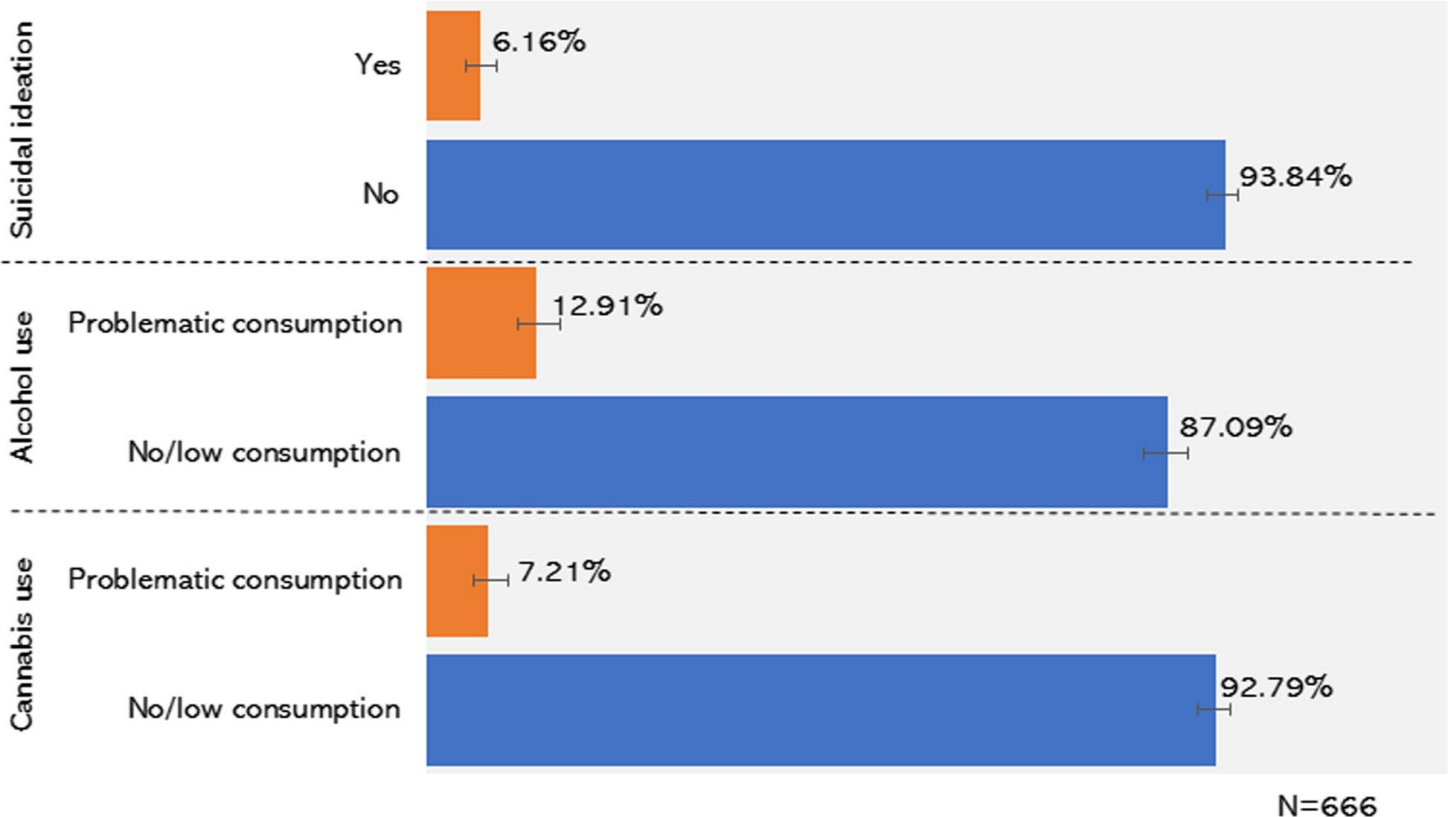
Percentages of respondents by the outcome measures and their corresponding 95% confidence intervals

As shown in Fig. 1, 6.2% of the Saskatchewan respondents said that they had contemplated suicide since the onset of the pandemic. The prevalence of problematic alcohol consumption was 12.9%, and the prevalence of problematic cannabis use was 7.2%. The prevalence of dual-problematic use, cannabis and alcohol, was 4.7%.

**Fig. 2:**
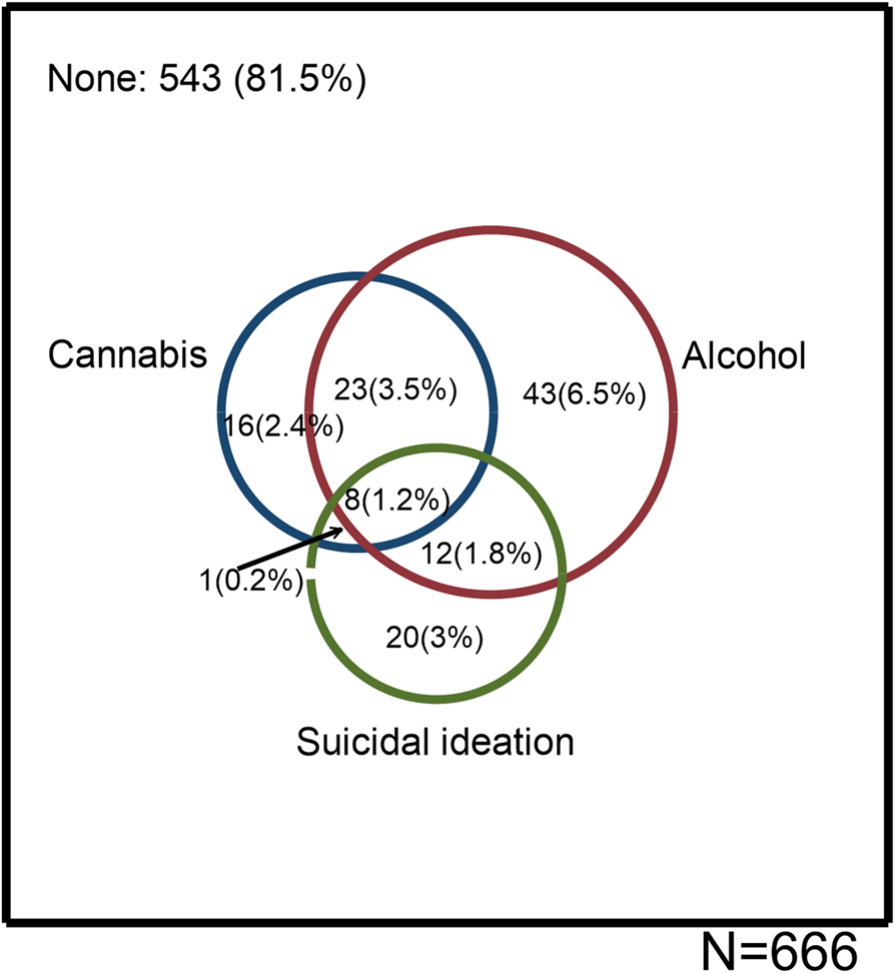
Intersectionality of suicidal ideation, problematic cannabis use and problematic alcohol use during COVID-19 pandemic. Circles are scaled to the frequency of respondents

Of the overall sample (666 respondents), 8 people (1.2%) indicated all three experiences: problematic alcohol and cannabis use and suicidal ideation (Fig. 2). In terms of two experiences, 12 (1.8%) indicated suicide ideation and problematic alcohol use, but only one respondent indicated suicide ideation and problematic cannabis use. Problematic alcohol use and problematic cannabis use were more prevalent, with 23 (3.5%) reporting this co-occurrence.

Problematic alcohol use was the most prevalent single experience reported, by 43 (6.5%) respondents. This was followed by suicide ideation (20 or 3%) and problematic cannabis use (16 or 2.4%).

Of 103 respondents who were users of at least one substance, 21 people (20.4%) had experienced suicidal thoughts. The prevalence of suicidal ideation among those who used alcohol (20 out of 86, or 23.2%) was more than those who used cannabis (9 out of 48 or 18.7%). As expected, the prevalence of suicidal ideation was higher among those who used both substances (8 out of 31 or 25.8%). Of 563 participants who neither experienced problematic alcohol use nor problematic cannabis use, 20 people (3.5%) reported suicidal ideation.

#### Prevalence of suicide ideation, problematic alcohol use, problematic cannabis use by correlates

The prevalence of suicidal ideation, problematic cannabis use, and alcohol use significantly differed by sociodemographic factors (see supplementary table 1, S1). The highest prevalence was seen among young people (16–34 years vs older): 17.4% for suicidal ideation, 22.1% for problematic cannabis use and 29.1% for problematic alcohol use; those who self-identified as BIPOC (vs white): 14.9% for suicidal ideation, 20.3% for problematic cannabis use and 29.7% for problematic alcohol use; and those identifying as LGBTQIA2S + (vs not): 17.1% for suicidal ideation, 25.7% for problematic cannabis use and 34.3% for problematic alcohol use.

#### Relationship between suicidal ideation, problematic cannabis use and problematic alcohol use

The unadjusted tetrachoric correlations show significant positive correlation between different pairs of outcomes: strongest for problematic cannabis use and problematic alcohol use (rho = 0.74, *p*-value < 0.001), modest for suicidal ideation and problematic alcohol use (rho = 0.56, *p*-value < 0.01), and weakest for suicidal ideation and problematic cannabis use (rho = 0.37, *p*-value = 0.004). As shown in Table 2, the tetrachoric correlation coefficients from the trivariate probit regression model changed after adjusting the confounding effects of predictor variables. Interestingly, problematic cannabis use was no longer significantly related with suicidality after accounting for the selected sociodemographic factors, mental health status, and adaptability factors. However, people who reported problematic alcohol use were likely to have experienced suicidal ideation, (rho = 0.46, *p*-value < 0.001). As expected, problematic cannabis and alcohol use were strongly correlated (rho = 0.65, *p*-value < 0.001).

**Table 2:** Predicted probabilities, goodness-of-fit, and correlation of the outcome measures

	Suicidal ideation	Problematic cannabis use	Problematic alcohol use
Marginal probability	0.068	0.073	0.134
Joint probability of occurrence of all events (success)	0.012		
Joint probability of failure of all events	0.805		
Correlation coefficient (suicidal ideation and problematic cannabis use)	0.20		
Correlation coefficient (suicidal ideation and problematic alcohol use)	0.46***		
Correlation coefficient (problematic alcohol and cannabis use)	0.65***		
Number of draws	26		
Number of observations	556		
AIC	792.62		
BIC	974.09		
Log likelihood	-354.31		
Wald Chi2	149.58		
Prob > Chi2	< 0.001		

Likelihood ratio test of rho21 = rho31 = rho32 = 0: Chi2(3) = 43.17, *p*-value < 0.001; where 1 = suicidal ideation, 2 = cannabis use and 3 = alcohol use

*AIC* Akaike Information Criteria, *BIC* Bayesian Information Criteria

^***^p < 0.001

After accounting for the unobservable correlation errors from the dependent variables and the confounding effects of the independent variables, the probability of individuals to jointly experience suicidal ideation, and problematic cannabis and alcohol use was 1.2%, compared to their failure of 80.5% to jointly experience the three outcomes of interest. The marginal or unconditional probability of problematic alcohol use was higher (13.4%), than the probability of problematic cannabis use (7.3%), and suicidal ideation (6.8%).

The log likelihood statistic (-354.31) and Wald Chi-square statistic (149.58, *p-value* < 0.001) indicate that the variables included in the final model jointly explained the probability of suicidal ideation, problematic cannabis and alcohol use; the model goodness-of-fit is deemed satisfactory. In addition, the likelihood ratio test of independence of error terms is significant (Chi2=43.17, *p-value* < 0.001, further justifying the use of a trivariate probit for the unbiased estimates.

#### Factors associated with suicidal ideation, problematic cannabis and alcohol use

**Fig. 3:**
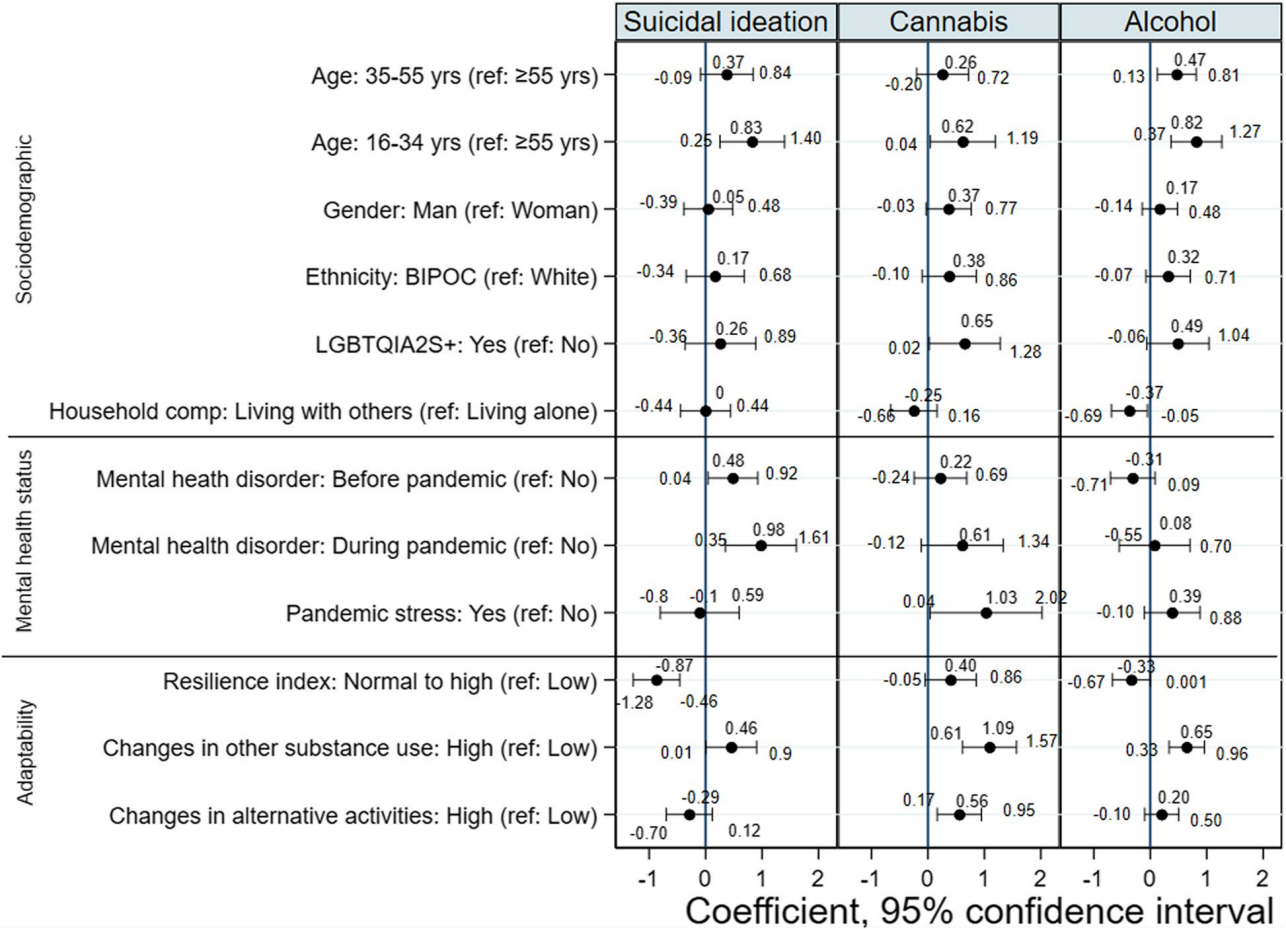
Coefficients and 95% confidence intervals from the multivariable trivariate probit regression. The point estimate (magnitude of effect) is represented by a small bullet, and confidence intervals by the horizontal lines, with each end of the lines representing the boundaries. The point estimates that are on the positive side of zero show harmful effects, and those on the negative side show protective effects. There are no statistically significant association where the confidence intervals crossed the zero (line of null effect), but where the confidence intervals did not cross zero, statistically significant association can be inferred

**Fig. 4:**
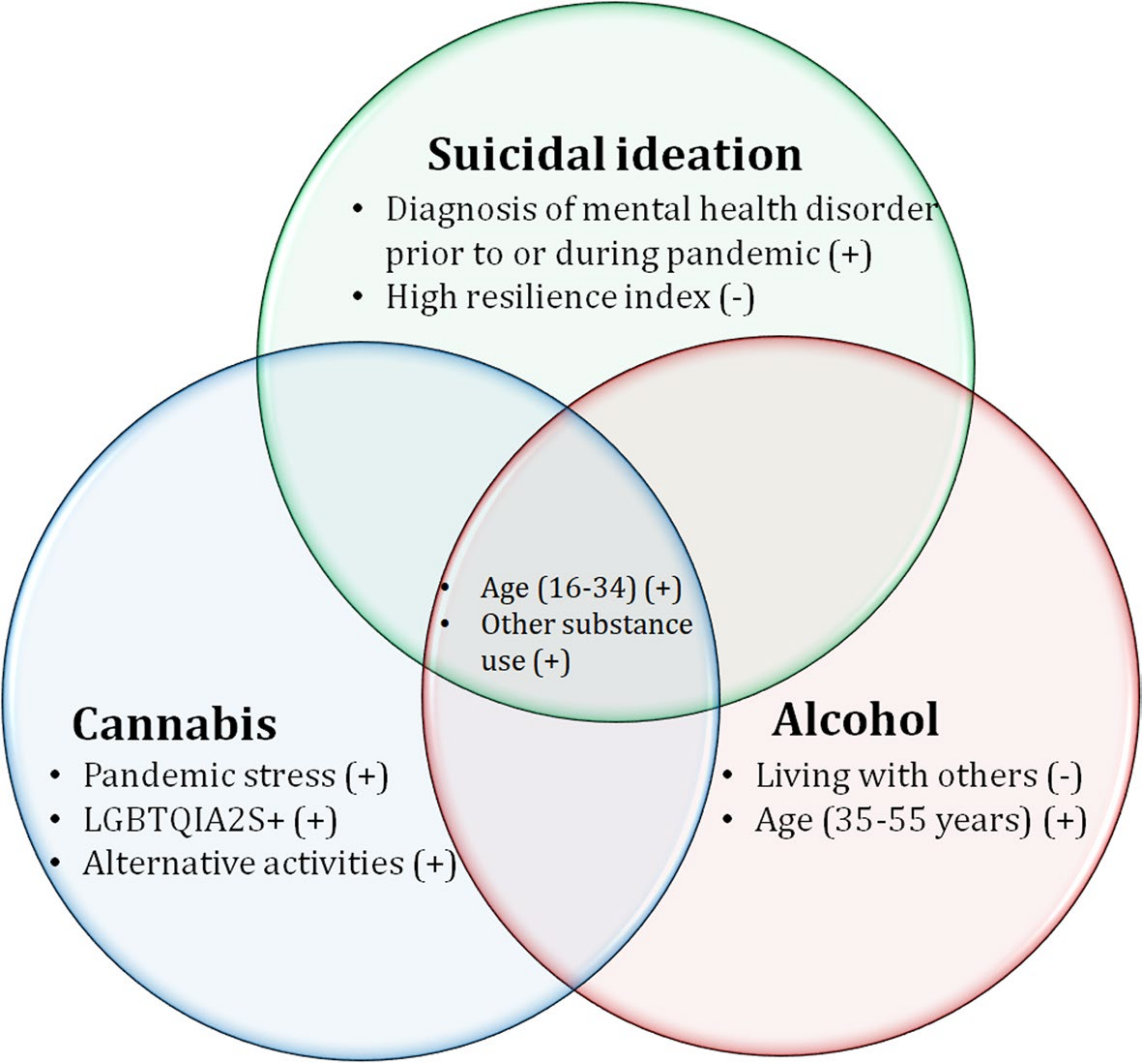
Summary factors significantly associated with suicidal ideation, problematic cannabis use and alcohol use during COVID-19 pandemic in Saskatchewan, March 2022

The coefficients and their 95% confidence intervals of the trivariate probit regression are presented in Fig. 3 (full model). Figure 4 presents a summary of the significantly associated factors with the three outcomes. Younger respondents,16–34 years, and those who scored high on the changes in other substance use had higher likelihood of all three adverse outcomes: suicidality, problematic cannabis use and alcohol use. Compared to those who were 55 years or older, those who were in the 16–34 age bracket had 82.7% higher probability of having suicidal thoughts, 61.8% higher probability of problematic cannabis use and 81.7% higher probability of problematic alcohol use.

The probabilities of suicidal ideation (45.6%), problematic cannabis use (109.2%) and problematic alcohol use (64.7%) were higher among respondents who scored higher on the changes in other substance use controlling for other variables presented in the model. For cannabis use only, and not for the two other outcomes, those who scored higher in the changes in alternative activities had 55.7% higher probability of having problems with use of this substance.

COVID-19 related stress was not significantly associated with suicidality and problematic alcohol use. However, pandemic stress increased the probability of problematic cannabis use by 103%. Also, the probability of problematic cannabis use among those who self-identified as LGBTQIA2S + was higher than among those who did not (Fig. 3). Having a diagnosis of mental health disorders, either before or during the pandemic, increased the probability of suicidal thoughts by 48.3% and 98%, respectively. Respondents who indicated they were more likely to bounce back after the pandemic is over (i.e., normal to high resiliency group vs low), had lower probability of suicidal ideation (86.9%). Furthermore, the probability of problematic alcohol use dropped by 36.8% among people who shared houses with others compared to those living alone.

## Discussion

This study reveals that the prevalence of multiple adverse mental health and behavioral issues, namely suicidal ideation, problematic use of alcohol and cannabis, among a sample of Saskatchewan residents is relatively high during the second year of the COVID-19 pandemic. We found that among people with problematic use of alcohol and cannabis, suicidal ideation was 26%, meaning that among this group one in four had thoughts of ending their lives during the pandemic. Suicidal ideation was more common among respondents who had problem with alcohol use than among people with problematic cannabis use. Younger respondents (16–34 years) and those reported recent changes in other substance use (e.g., use of other psychoactive drugs) were independent factors that were associated with the common experience of suicide ideation, problematic cannabis and alcohol use.

Some factors were associated with one outcome but not all three. Having a diagnosis of mental health disorders either before or during the pandemic, and the perceived inability to bounce back after the pandemic (low resilience) are strong correlates of suicidal ideation. Those who lived alone, between 35 and 55 years of age were more likely to report problematic alcohol use. Those who reported changes in alternative activities (such as physical and relaxation activities, and connection with social networks), who reported pandemic stress, and declared a LGBTQIA2S + identity had higher probability of problematic cannabis use.

The prevalence of problematic use of alcohol or cannabis during the COVID-19 pandemic reported here are comparable with the national prevalence of 14% and 8%, respectively (Table S2). Two years into the pandemic, in March 2022, 6.2% of Saskatchewan respondents have contemplated suicide compared to 3.3% in 2019—an increase of 87.9%—and 5.6% in Spring 2021, an increase of 10.7% [37].

Although, there is generally an increasing trend in suicidal ideation in Canada [37], the Saskatchewan prevalence is lower than the national prevalence of 8.1%. The Saskatchewan suicide rates have been consistently declining since 2018, reducing by 14% from 2018 to 2019 (20.7 deaths per 100,000 population to 17.8 deaths per 100,000 population) [8]. Moreover, the suicide rate has been documented to further drop by 22% from the 2019 to 2021 (13.8 deaths per 100,000 people in 2021) [8]. The reason the suicide rate in Saskatchewan showed improvement, while suicidal ideation did not, is not clear. A plausible explanation for this finding is that suicidal ideation varies in intensity, and duration; a few people might have proceeded with their suicide intentions and plans after the initial suicidal thoughts (low rates of active suicidal ideation). However, it should be noted that regardless of whether a suicidal thought is followed up by an attempt or not, it is still a mental health emergency because it is an early precursor to later attempted suicide (especially in the face of elevated rates of anxiety and depression). Given that active suicidal ideation (suicidal thoughts with plans to ending one’s life) is a more severe form of suicidal ideation than passive suicidal ideation (suicidal thoughts with no plans to end one’s life), this study could not delineate suicidal ideation by types. Further research is recommended to specifically estimate the magnitude and progression of suicidal ideation by types, and whether accessibility to social support and suicidal prevention programmes somehow reduce suicide rates.

While prevention efforts are unlikely to result in immediate changes in suicide rates, provincial efforts to increase prevention activities may be lowering the intensity of suicidal ideation from active to passive. The prevention activities were developed within the provincial sociocultural context, while leveraging community needs and strengths, as emphasized in the 2020 Saskatchewan Suicide Prevention Plan, commonly known as the “Pillars for Life” plan [56]. This plan aligns with the Saskatchewan’s Mental Health and Addictions Action Plan, and both plans recognize the importance of community driven and culturally responsive multisectoral collaborations [56, 57]. Through the continuous implementation of community-led initiatives such as the “Roots of Hope programmes” by the community gatekeepers in La Ronge, Meadow Lake, and Buffalo Narrows, public health awareness about suicide prevention, drug safety, and other mental health services are delivered in the communities [57]. In addition, crisis services, community recovery teams, police and crisis teams (PCT), and mental health capacity-building initiatives are being expanded within the province [57]. Despite the recent progress achieved in reducing suicide rates, the high rates of suicidal ideation are concerning. Also, there have been mixed reactions due to the recent budget cut for suicide prevention programmes by the government of Saskatchewan [58].

Consistent with other studies, suicidal ideation and problematic alcohol use are interrelated problems [59–61]. Alcohol and cannabis use coexist [62], as well, thus interventions aimed at improving problematic alcohol use are more likely to have reciprocal effects on both suicidal ideation and problematic cannabis use, and vice versa. Somewhat paradoxically but consistent with the findings of Zhang et al., [63] and Price et al., [16] we found no significant relationship between problematic cannabis use and suicidal ideation after adjusting for confounding effects of the selected covariables. Further studies are needed to clarify the association between problematic cannabis use and suicidal ideation.

In this study, the respondents who said they were having suicidal thoughts and problematic cannabis and alcohol use tend to be younger (16–34 years old) and were more likely to use other illicit and psychotropic substances, as indicative of their reported recent changes in other substance use. These findings are in line with shifting trends that were exacerbated by the pandemic. Before the pandemic, there was clustering of suicidal ideation at older age (≥ 65 years) [64, 65]. A longitudinal study conducted during the early months of the pandemic in the United States had established that young people were disproportionately affected by suicidal ideation partly because of substance use [66]. Also, more recent studies have noted the demographic shift in suicidal ideation from older population to younger population [37, 67]. The relationship between suicidal ideation, and experience with alcohol or cannabis use has been linked to other substance use such as polysubstance use [35].

This study found evidence that problematic alcohol use decreases with increasing age. Judging by the current data, this finding might indicate that young people used substances as part of the measures taken to ease pains and losses arising from the pandemic. For example, in our further analysis, we observed that more young people lost their jobs than other age groups (16–34 years old: 4%, 35–54 years old: 1% and ≥ 55 years old: 2%), which could have possibly increased economic hardship and solitude.

We also observed substance-specific correlates of problematic alcohol and cannabis use. Living arrangements have been shown to increase the risk of problematic alcohol use [68]. According to Joutsenniemi et al., [68] living with other people (social capital) promotes healthy lifestyle such as abstinence or low alcohol consumption by bolstering social cohesion and support. This association is broadly in line with our findings which shows that people who lived with others are less likely to experience problematic alcohol use. Alternatively, people who excessively drink alcohol may have problems maintaining relationships with other people. However, in line with the ideas of Canham et al., [69] and Rosenquist et al., [70] it is important to note the residual effects of shared ideologies among social groups on drinking behaviors of the respondents as some individuals might not drink alcohol because their friends were not, while others would drink due to interpersonal influence or pressure.

Consistent with literature [71–73], this study highlights a higher susceptibility of respondents who declared as LGBTQIA2S + , and who reported links between pandemic-related stress and problematic cannabis use. In this study, most of the respondents reported unstable financial situation (20%) as their biggest source of COVID-19 related stress. While some people might have consumed cannabis recreationally, according to the sexual minority stress theory [71–73], LGBTQIA2S + often experience higher level of stress, conflicts within household, social stigma, prejudice and social isolation which, in turn, could have facilitated problematic cannabis use. These experiences by the sexual minority groups could have been aggravated by the pandemic or persistent homophobic and transphobic culture in Canada [74–76].

Another important finding of this study is the elevated probability of suicidal ideation among individuals who reported they would have a difficult time bouncing back from the pandemic (low resiliency) and those diagnosed with mental health disorders, being stronger when diagnosed during the pandemic. Although it is still unclear whether a causal relationship exists, there continues to be growing body of evidence that mental health disorders (especially psychological distress and depression) are among the common risk factors of suicidality [77]. Early identification and treatment of mental health disorders can prevent suicidal ideation. While it is encouraging that 76% of the respondents reported their ability to bounce back after the pandemic, the probability of having suicidal thoughts was 87% higher among the respondents who assessed their resilience as low. This suggests that perceived resilience could be protective against suicide during the pandemic [78–80]. In the midst of the crisis, helping people to build resilience through social support and teaching them about behaviors that promotes healthy lifestyle may reduce pandemic-related stress and suicidal behavior, and therefore should be an integral part of suicide prevention measures.

This study contributes by advancing our understanding of the interplay between suicidal ideation, problematic use of alcohol, and cannabis use in the second year of the COVID-19 pandemic, and establishing the roles of sociodemographic, mental health status and adaptability factors. Using separate models, previous studies have failed to address the correlation between the three outcome variables, with a possibility of introducing type-1 errors (i.e., concluding that results are statistically significant when they are not). To address the correlation structure and generate unbiased estimates, this study utilized trivariate probit regression to simultaneously predict the outcomes of interest.

There are some potential limitations to consider. First, participants’ responses were self-reported and might be subject to recall bias. Using validated and calibrated tools might have reduced this risk. Second, non-probabilistic sampling (quota sampling) was employed, so not every Saskatchewan adult had an equal chance of being recruited into the study. However, considering that sampling was done based on geographic distribution, age, gender, immigration status and ethnicity, the composition of study sample is representative of the Saskatchewan adult population. Lastly, causal inferences can not be made because of the cross-sectional nature of the study. Our findings should therefore be interpreted in the light of associations.

In summary, suicidality among Saskatchewan adults is not occurring in isolation but occur simultaneously with problematic alcohol use. This finding suggests that recognition of early warning signs and treatment of individuals with problematic alcohol use are important for suicide prevention. Also, those who reported problematic alcohol use are more likely to experience problematic cannabis use, although cannabis use did not have an independent association with suicidal ideation. This study also provides a motivation for implementing age-responsive interventions and public education on coping behaviors because these factors are shared among people living with suicidal ideation, problematic alcohol, and cannabis use. The distinct roles of mental health disorder and resilience in reducing suicidality during crisis (as was the case in this study, COVID-19 pandemic) deserve attention by the stakeholders. Mental health supports should be intensified in the communities. Also, treatment and prevention of cannabis dependence and its attendant problems among LGBTQIA2S + and other people affected by the pandemic is recommended and considered as good use of public health resources. Given the extent to which people who lived alone were at risk of problematic alcohol use, these groups should be prioritized. As the pandemic persists, it is necessary to deliver screening and educational interventions to the high-risk individuals identified in this study.

### Supplementary Information

**Additional file 1: Table A1.** Recent changes in coping behavior, MHCC-CCSA Survey. **Table A2.** Eigenvalues of the principal factor analysis of the retained11 items (after dropping items with factor loading <0.3). **Table A3.** Rotated factor loadings (pattern matrix) and unique variances of the retained items (based on absolute loading of 0.3). **Table A4.** Kaiser-Meyer-Olkin measure of sampling adequacy. **Figure A1.** Correlations of the 17 items from the coping behavior scale. **Figure A2.** Scree plot of eigenvalues after factor analysis. **Figure A3.** Score plot of the retained factors. **Figure A4.** Loading plot of the retained factors and items

**Additional file 2: Table S1.** Prevalence of suicidal ideation, and problematic cannabis and alcohol use. **Tables S2.** Prevalence of suicidal ideation, problematic cannabis and alcohol use, Canada and Saskatchewan province

## Data Availability

The datasets used for this study are owned by Mental Health Commission of Canada (MHCC) and Canadian Centre on Substance Use and Addiction (CCSA), and shared with the authors under a MHCC-CCSA data sharing agreement with NM. The datasets generated and/or analysed during the current study are available from the corresponding author on reasonable request. As required by the data management policy, the dataset for this study is available from the MHCC-CCSA survey team on an official written request.
