# Supplementary material for: Prevalence and factors associated with suicidal ideation, cannabis, and alcohol use during the COVID-19 pandemic in Saskatchewan: findings from a joint-effect modeling
Source: BMC Psychiatry. 2023 Aug 8;23:571. doi: 10.1186/s12888-023-05051-w (PMC10408153; doi:10.1186/s12888-023-05051-w)
Supplement: Supplementary file 1 — Additional file 1: Table A1. Recent changes in coping behavior, MHCC-CCSA Survey. Table A2. Eigenvalues of the principal factor analysis of the retained11 items (after dropping items with factor loading <0.3). Table A3. Rotated factor loadings (pattern matrix) and unique variances of the retained items (based on absolute loading of 0.3). Table A4. Kaiser-Meyer-Olkin measure of sampling adequacy. Figure A1. Correlations of the 17 items from the coping behavior scale. Figure A2. Scree plot of eigenvalues after factor analysis. Figure A3. Score plot of the retained factors. Figure A4. Loading plot of the retained factors and items [file 12888_2023_5051_MOESM1_ESM.docx]

Appendix A

**Prevalence and factors associated with suicidal ideation, cannabis, and alcohol use during the COVID-19 pandemic in Saskatchewan: findings from a joint-effect modeling**

*Running title: Suicidal ideation, cannabis, and alcohol use in Saskatchewan*

Daniel A. Adeyinka^1,2^, Nuelle Novik^1,3^, Gabriela Novotna^1,3^, Mary Bartram^4,5^, Robert Gabrys^6^, Nazeem Muhajarine^1,2^

**Affiliations and Addresses:**

^1^Saskatchewan Population Health and Evaluation Research Unit (SPHERU), University of Saskatchewan, 104 Clinic Place, Saskatoon, SK, S7N 2Z4

^2^Department of Community Health and Epidemiology, College of Medicine, University of Saskatchewan, 107 Wiggins Rd, Saskatoon, SK, S7N 5E5

^3^Faculty of Social Work, University of Regina, 3737 Wascana Parkway, Regina, SK, S4S 0A2

^4^School of Public Health and Administration, Carleton University, Ottawa, ON, K1S 5B6

^5^Mental Health Commission of Canada, Ottawa ON, K1R 1A4

^6^Canadian Centre on Substance Use and Addiction, Ottawa, ON, K1P 5E7

**Corresponding Author: Nazeem Muhajarine, e: nazeem.muhajarine@usask.ca**

Table A1: Recent changes in coping behavior, MHCC-CCSA Survey

**Self care/coping: During the past month**, have you engaged in **more or less** of the following activities?

|  | **List** | I do not engage in this activity | Less | The same | More |
| --- | --- | --- | --- | --- | --- |
| **Item** | **Value** | 1 | 2 | 3 | 4 |
| Connecting with friends and family through technology (e.g., Facetime) | 1 | 🔿 | 🔿 | 🔿 | 🔿 |
| Walking/jogging | 2 | 🔿 | 🔿 | 🔿 | 🔿 |
| Exercising | 3 | 🔿 | 🔿 | 🔿 | 🔿 |
| Online streaming channels (e.g., Netflix) | 4 | 🔿 | 🔿 | 🔿 | 🔿 |
| Time with pet | 5 | 🔿 | 🔿 | 🔿 | 🔿 |
| Eating food | 6 | 🔿 | 🔿 | 🔿 | 🔿 |
| Mindfulness (e.g., meditation) | 7 | 🔿 | 🔿 | 🔿 | 🔿 |
| Social media | 8 | 🔿 | 🔿 | 🔿 | 🔿 |
| Watching or reading the news | 9 | 🔿 | 🔿 | 🔿 | 🔿 |
| Relaxation activities (e.g., bath, hot tub) | 10 | 🔿 | 🔿 | 🔿 | 🔿 |
| Shopping (online or in-store) | 11 | 🔿 | 🔿 | 🔿 | 🔿 |
| Working more | 12 | 🔿 | 🔿 | 🔿 | 🔿 |
| Sexual activity | 13 | 🔿 | 🔿 | 🔿 | 🔿 |
| Drinking alcohol (beverages/drinks)* | 14 | 🔿 | 🔿 | 🔿 | 🔿 |
| Consuming cannabis * | 15 | 🔿 | 🔿 | 🔿 | 🔿 |
| Smoking cigarettes | 16 | 🔿 | 🔿 | 🔿 | 🔿 |
| Using e-cigarettes (e.g., vape pen) | 17 | 🔿 | 🔿 | 🔿 | 🔿 |
| Taking prescribed psychoactive drugs (e.g., Ativan, Xanax, Dilaudid, etc.) – excluding cannabis) | 18 | 🔿 | 🔿 | 🔿 | 🔿 |
| Taking other illegal psychoactive drugs (e.g., cocaine, opioids, meth, etc.) | 19 | 🔿 | 🔿 | 🔿 | 🔿 |

*Items 14 and 15 were excluded from the factor analysis because they are part of the outcome factors

Table A2: Eigenvalues of the principal factor analysis of the retained11 items (after dropping items with factor loading <0.3)

|  | Unrotated | | | | Orthogonal varimax rotation* | | | |
| --- | --- | --- | --- | --- | --- | --- | --- | --- |
| Factor | Eigenvalue | Difference | Proportion | Cumulative | Eigenvalue | Difference | Proportion | Cumulative |
| Factor1 | 2.19011 | 0.90434 | 0.7506 | 0.7506 | 1.81091 | 0.14594 | 0.6206 | 0.6206 |
| Factor2 | 1.28577 | 1.10212 | 0.4407 | 1.1912 | 1.66497 | . | 0.5706 | 1.1912 |
| Factor3 | 0.18365 | 0.04302 | 0.0629 | 1.2542 |  | | | |
| Factor4 | 0.14063 | 0.12271 | 0.0482 | 1.3024 |  |  |  |  |
| Factor5 | 0.01792 | 0.07924 | 0.0061 | 1.3085 |  |  |  |  |
| Factor6 | -0.06133 | 0.03993 | -0.021 | 1.2875 |  |  |  |  |
| Factor7 | -0.10126 | 0.0159 | -0.0347 | 1.2528 |  |  |  |  |
| Factor8 | -0.11716 | 0.05933 | -0.0402 | 1.2126 |  |  |  |  |
| Factor9 | -0.17648 | 0.02633 | -0.0605 | 1.1522 |  |  |  |  |
| Factor10 | -0.20281 | 0.03835 | -0.0695 | 1.0827 |  |  |  |  |
| Factor11 | -0.24117 | . | -0.0827 | 1 |  |  |  |  |

*Kaiser normalization

Table A3: Rotated factor loadings (pattern matrix) and unique variances of the retained items (based on absolute loading of 0.3)

| Item | Factor1 | Factor2 | Uniqueness |
| --- | --- | --- | --- |
| Cronbach’s alpha | 0.7719 | 0.6533 |  |
| Connecting with friends and family through technology (e.g., Facetime) |  | 0.3432 | 0.8628 |
| Walking/jogging |  | 0.6723 | 0.5478 |
| Exercising |  | 0.6517 | 0.5753 |
| Mindfulness (e.g., meditation) |  | 0.4289 | 0.7829 |
| Relaxation activities (e.g., bath, hot tub) |  | 0.4111 | 0.8306 |
| Working more |  | 0.3085 | 0.9048 |
| Sexual activity |  | 0.4049 | 0.825 |
| Smoking cigarettes | 0.5784 |  | 0.6636 |
| Using e-cigarettes (e.g., vape pen) | 0.7295 |  | 0.4516 |
| Taking prescribed psychoactive drugs (e.g., Ativan, Xanax, Dilaudid, etc.) – excluding cannabis) | 0.5933 |  | 0.6228 |
| Taking other illegal psychoactive drugs (e.g., cocaine, opioids, meth, etc.) | 0.7265 |  | 0.4569 |

Table A4: Kaiser-Meyer-Olkin measure of sampling adequacy

| Item | KMO |
| --- | --- |
| Connecting with friends and family through technology (e.g., Facetime) | 0.7949 |
| Walking/jogging | 0.6798 |
| Exercising | 0.6776 |
| Mindfulness (e.g., meditation) | 0.8335 |
| Relaxation activities (e.g., bath, hot tub) | 0.8117 |
| Working more | 0.7085 |
| Sexual activity | 0.7997 |
| Smoking cigarettes | 0.8181 |
| Using e-cigarettes (e.g., vape pen) | 0.7533 |
| Taking prescribed psychoactive drugs (e.g., Ativan, Xanax, Dilaudid, etc.) – excluding cannabis) | 0.8235 |
| Taking other illegal psychoactive drugs (e.g., cocaine, opioids, meth, etc.) | 0.7642 |
| Overall | 0.7610 |

Figure A1: Correlations of the 17 items from the coping behavior scale

Figure A2: Scree plot of eigenvalues after factor analysis

Figure A3: Score plot of the retained factors

Figure A4: Loading plot of the retained factors and items
