# Supplementary material for: Prevalence and factors associated with suicidal ideation, cannabis, and alcohol use during the COVID-19 pandemic in Saskatchewan: findings from a joint-effect modeling
Source: BMC Psychiatry. 2023 Aug 8;23:571. doi: 10.1186/s12888-023-05051-w (PMC10408153; doi:10.1186/s12888-023-05051-w)
Supplement: Supplementary file 2 — Additional file 2: Table S1. Prevalence of suicidal ideation, and problematic cannabis and alcohol use. Tables S2. Prevalence of suicidal ideation, problematic cannabis and alcohol use, Canada and Saskatchewan province [file 12888_2023_5051_MOESM2_ESM.docx]

**Supplementary Table**

**Prevalence and factors associated with suicidal ideation, cannabis, and alcohol use during the COVID-19 pandemic in Saskatchewan: findings from a joint-effect modeling**

*Running title: Suicidal ideation, cannabis, and alcohol use in Saskatchewan*

Daniel A. Adeyinka^1,2^, Nuelle Novik^1,3^, Gabriela Novotna^1,3^, Mary Bartram^4,5^, Robert Gabrys^6^, Nazeem Muhajarine^1,2^

**Affiliations and Addresses:**

^1^Saskatchewan Population Health and Evaluation Research Unit (SPHERU), University of Saskatchewan, 104 Clinic Place, Saskatoon, SK, S7N 2Z4

^2^Department of Community Health and Epidemiology, College of Medicine, University of Saskatchewan, 107 Wiggins Rd, Saskatoon, SK, S7N 5E5

^3^Faculty of Social Work, University of Regina, 3737 Wascana Parkway, Regina, SK, S4S 0A2

^4^School of Public Health and Administration, Carleton University, Ottawa, ON, K1S 5B6

^5^Mental Health Commission of Canada, Ottawa ON, K1R 1A4

^6^Canadian Centre on Substance Use and Addiction, Ottawa, ON, K1P 5E7

**Corresponding Author: Nazeem Muhajarine, e: nazeem.muhajarine@usask.ca**

Table S1. Prevalence of suicidal ideation, and problematic cannabis and alcohol use

| **Variables** | **Overall** | **Suicidal ideation** | | **Problematic cannabis use** | | **Problematic alcohol use** | |
| --- | --- | --- | --- | --- | --- | --- | --- |
|  | n (%) | *No (n,%)* | *Yes (n,%)* | *No (n,%)* | *Yes (n,%)* | *No (n,%)* | *Yes (n,%)* |
| **Sociodemographic** | | | | | | | |
| *Age (years) (n=666)* | | | | | | | |
| 16-34 | 86 (12.91) | 71 (82.56) | 15 (17.44) | 67 (77.91) | 19 (22.09) | 61 (70.93) | 25 (29.07) |
| 35-54 | 189 (28.38) | 173 (91.53) | 16 (8.47) | 174 (92.06) | 15 (7.94) | 157 (83.07) | 32 (16.93) |
| ≥55 | 391 (58.71) | 381 (97.44) | 10 (2.56) | 377 (96.42) | 14 (3.58) | 362 (92.58) | 29 (7.42) |
| P-value |  | <0.001 | | <0.001 | | <0.001 | |
| *Gender (n=663)* | | | | | | | |
| Woman | 378 (57.01) | 349 (92.33) | 29 (7.67) | 357 (94.44) | 21 (5.56) | 331 (87.57) | 47 (12.43) |
| Man | 285 (42.99) | 273 (95.79) | 12 (4.21) | 259 (90.88) | 26 (9.12) | 247 (86.67) | 38 (13.33) |
| P-value |  | 0.067 | | 0.076 | | 0.732 | |
| *Highest education (n=663)* | | | | | | | |
| High school or less | 191 (28.81) | 180 (94.24) | 11 (5.79) | 177 (92.67) | 14 (7.33) | 166 (86.91) | 25 (13.09) |
| College | 254 (38.31) | 236 (92.91) | 18 (7.09) | 239 (94.09) | 15 (5.91) | 226 (88.98) | 28 (11.02) |
| University | 218 (32.88) | 206 (94.5) | 12 (5.5) | 199 (91.28) | 19 (8.72) | 185 (84.86) | 33 (15.14) |
| P-value |  | 0.745 | | 0.501 | | 0.414 | |
| *Household income (n=585)* | | | | | | | |
| ≤ $20k | 37 (6.32) | 33 (89.19) | 4 (10.81) | 32 (86.49) | 5 (13.51) | 31 (83.78) | 6 (16.22) |
| $21k-$50k | 166 (28.38) | 152 (91.57) | 14 (8.43) | 151 (90.96) | 15 (9.04) | 144 (86.75) | 22 (13.25) |
| $51k-$100k | 235 (40.17) | 224 (95.32) | 11 (4.68) | 222 (94.47) | 13 (5.53) | 203 (86.38) | 32 (13.62) |
| >$100k | 147 (25.13) | 138 (93.88) | 9 (6.12) | 136 (92.52) | 11 (7.48) | 127 (86.39) | 20 (13.61) |
| P-value |  | 0.268* | | 0.26* | | 0.973 | |
| *Ethnicity (n=654)* | | | | | | | |
| White | 580 (88.69) | 550 (94.83) | 30 (5.17) | 547 (94.31) | 33 (5.69) | 516 (88.97) | 64 (11.03) |
| BIPOC | 74 (11.31) | 63 (85.14) | 11 (14.86) | 59 (79.73) | 15 (20.27) | 52 (70.27) | 22 (29.73) |
| P-value |  | 0.004 | | <0.001 | | <0.001 | |
| *LGBTQIA2S+ (n=659)* | | | | | | | |
| No | 624 (94.69) | 590 (94.55) | 34 (5.45) | 585 (93.75) | 39 (6.25) | 552 (88.46) | 72 (11.54) |
| Yes | 35 (5.31) | 29 (82.86) | 6 (17.14) | 26 (74.29) | 9 (25.71) | 23 (65.71) | 12 (34.29) |
| P-value |  | 0.015* | | <0.001* | | 0.001* | |
| *Household composition (n=664)* | | | | | | | |
| Living alone | 174 (26.2) | 163 (93.68) | 11 (6.32) | 156 (89.66) | 18 (10.34) | 145 (83.33) | 29 (16.67) |
| Living with others | 490 (73.8) | 460 (93.88) | 30 (6.12) | 460 (93.88) | 30 (6.12) | 433 (88.37) | 57 (11.63) |
| P-value |  | 0.925 | | 0.065 | | 0.089 | |
| *Migration status (n=664)* | | | | | | | |
| Canadian-born | 595 (89.61) | 560 (94.12) | 35 (5.88) | 554 (93.11) | 41 (6.89) | 523 (87.90) | 72 (12.1) |
| Immigrants | 69 (10.39) | 64 (92.75) | 5 (7.25) | 63 (91.3) | 6 (6.87) | 56 (81.16) | 13 (18.84) |
| P-value |  | 0.595* | | 0.617* | | 0.1133 | |
| *Place of residence (n=514)* | | | | | | | |
| Regina | 177 (34.44) | 168 (94.92) | 9 (5.08) | 166 (93.79) | 11 (6.21) | 160 (90.4) | 17 (9.6) |
| Saskatoon | 197 (38.33) | 187 (94.92) | 10 (5.08) | 180 (91.37) | 17 (8.63) | 170 (86.29) | 27 (13.71) |
| North | 46 (8.95) | 40 (86.96) | 6 (13.04) | 44 (95.65) | 2 (4.35) | 39 (84.78) | 7 (15.22) |
| Central | 36 (7) | 35 (97.22) | 1 (2.78) | 35 (97.22) | 1 (2.78) | 31 (86.11) | 5 (13.89) |
| South | 58 (11.28) | 56 (96.55) | 2 (3.45) | 56 (96.55) | 2 (3.45) | 52 (89.66) | 6 (10.34) |
| P-value |  | 0.275* | | 0.604* | | 0.651* | |
| **Mental health status** | | | | | | | |
| *Mental health disorder (n=658)* | | | | | | | |
| No | 494 (75.08) | 479 (96.96) | 15 (3.04) | 469 (94.94) | 25 (5.06) | 437 (88.46) | 57 (11.54) |
| Before pandemic | 140 (21.28) | 123 (87.86) | 17 (12.14) | 126 (90) | 14 (10) | 122 (87.14) | 18 (12.86) |
| During pandemic | 24 (3.65) | 16 (66.67) | 8 (33.33) | 16 (66.67) | 8 (33.3) | 16 (66.67) | 8 (33.33) |
| P-value |  | <0.001* | | <0.001* | | 0.015* | |
| *Pandemic stress (n=644)* | | | | | | | |
| No | 108 (16.77) | 106 (98.15) | 2 (1.85) | 107 (99.07) | 1 (0.93) | 103 (95.37) | 5 (4.63) |
| Yes | 536 (83.23) | 497 (92.72) | 39 (7.28) | 490 (91.42) | 46 (8.58) | 457 (85.26) | 79 (14.74) |
| P-value |  | 0.035 | | 0.005 | | 0.004 | |
| **Adaptability** | | | | | | | |
| *Resilience (n=665)* | | | | | | | |
| Low (1-2.99) | 162 (24.36) | 134 (82.72) | 28 (17.28) | 151 (93.21) | 11 (6.79) | 128 (79.01) | 34 (20.99) |
| Normal to high (3-5) | 503 (75.64) | 490 (97.42) | 13 (2.58) | 466 (92.64) | 37 (7.36) | 451 (89.66) | 52 (10.34) |
| P-value |  | <0.001 | | 0.809 | | <0.001 | |
| *Recent changes in other substance use (median, n=598)* | | | | | | | |
| Low (-0.69 to -0.36) | 300 (50.17) | 293 (97.67) | 7 (2.33) | 296 (98.67) | 4 (1.33) | 283 (94.33) | 17 (5.67) |
| High (-0.36 to 3.86) | 298 (49.83) | 266 (89.26) | 32 (10.72) | 258 (86.58) | 40 (13.42) | 236 (79.19) | 62 (20.81) |
| P-value |  | 0.001 | | <0.001 | | <0.001 | |
| *Changes in alternative activities (median, n=598)* | | | | | | | |
| Low (-2.31 to 0.09) | 299 (50) | 273 (91.30) | 26 (8.70) | 283 (94.65) | 16 (5.35) | 260 (86.96) | 39 (13.04) |
| High (0.09 to 2.06) | 299 (50) | 286 (95.65) | 13 (4.35) | 271 (90.64) | 28 (9.36) | 259 (86.62) | 40 (13.38) |
| P-value |  | 0.031 | | 0.06 | | 0.904 | |
| **Access to formal treatment services** | | | | | | | |
| *Mental health services (n=642)* | | | | | | | |
| Not needed and not accessed | 479 (74.61) | 466 (97.29) | 13 (2.71) | 459 (95.82) | 20 (4.18) | 428 (89.35) | 51 (10.65) |
| Needed but not accessed | 111 (17.29) | 100 (90.09) | 11 (9.91) | 100 (90.09) | 11 (9.91) | 99 (89.19) | 12 (10.81) |
| Needed and accessed | 52 (8.1) | 38 (73.08) | 14 (26.92) | 42 (80.77) | 10 (19.23) | 38 (73.08) | 14 (26.92) |
| P-value |  | <0.001* | | <0.001* | | 0.003 | |
| *Cannabis addiction services (n=662)* | | | | | | | |
| Not applicable | 171 (25.83) | 166 (97.08) | 5 (2.92) | 168 (98.25) | 3 (1.75) | 150 (87.72) | 21 (12.28) |
| No | 479 (72.36) | 447 (93.32) | 32 (6.68) | 443 (92.48) | 36 (7.52) | 424 (88.52) | 55 (11.48) |
| Yes | 12 (1.81) | 8 (66.67) | 4 (33.33) | 3 (25) | 9 (75) | 3 (25) | 9 (75) |
| P-value |  | 0.001* | | <0.001* | | <0.001* | |
| *Alcohol addiction services (n=662)* | | | | | | | |
| Not applicable | 80 (12.08) | 79 (98.75) | 1 (1.25) | 79 (98.75) | 1 (1.25) | 77 (96.25) | 3 (3.75) |
| No | 565 (85.35) | 530 (93.81) | 35 (6.19) | 530 (93.81) | 35 (6.19) | 498 (88.14) | 67 (11.86) |
| Yes | 17 (2.57) | 13 (76.47) | 4 (23.53) | 6 (35.29) | 11 (64.71) | 2 (11.76) | 15 (88.24) |
| P-value |  | 0.006* | | <0.001* | | <0.001* | |

**Fisher's exact test, otherwise Chi-square test*

Tables S2: Prevalence of suicidal ideation, problematic cannabis and alcohol use, Canada and Saskatchewan province

| Prevalence (%) | Canada | Saskatchewan |
| --- | --- | --- |
| Suicidal ideation | 8.11 | 6.16 |
| Problematic alcohol use | 14.09 | 12.91 |
| Problematic cannabis use | 7.96 | 7.21 |
